# The Divergent Effects of CDPPB and Cannabidiol on Fear Extinction and Anxiety in a Predator Scent Stress Model of PTSD in Rats

**DOI:** 10.3389/fnbeh.2019.00091

**Published:** 2019-05-10

**Authors:** John Shallcross, Peter Hámor, Allison R. Bechard, Madison Romano, Lori Knackstedt, Marek Schwendt

**Affiliations:** ^1^Department of Psychology, University of Florida, Gainesville, FL, United States; ^2^Center for Addiction Research & Education, College of Medicine, University of Florida, Gainesville, FL, United States

**Keywords:** fear extinction, TMT, resilient, mGlu5, mPFC, BLA, Fos

## Abstract

Post-traumatic stress disorder (PTSD) currently has no FDA-approved treatments that reduce symptoms in the majority of patients. The ability to extinguish fear memory associations is impaired in PTSD individuals. As such, the development of extinction-enhancing pharmacological agents to be used in combination with exposure therapies may benefit the treatment of PTSD. Both mGlu5 and CB1 receptors have been implicated in contextual fear extinction. Thus, here we tested the ability of the mGlu5 positive allosteric modulator 3-Cyano-N-(1,3-diphenyl-1H-pyrazol-5-yl)benzamide (CDPPB) and cannabidiol (CBD) to reduce both conditioned and unconditioned fear. We used a predator-threat animal model of PTSD which we and others have previously shown to capture the heterogeneity of anxiety responses observed in humans exposed to trauma. Here, 1 week following a 10-min exposure to predator scent stress, rats were classified into stress-Susceptible and stress-Resilient phenotypes using behavioral criteria for elevated plus maze and acoustic startle response performance. Two weeks after classification, rats underwent 3 days of contextual fear extinction and were treated with vehicle, CDPPB or CBD prior to each session. Finally, the light-dark box test was employed to assess phenotypic differences and the effects of CDPPB and CBD on unconditioned anxiety. CDPBB but not CBD, reduced freezing in Susceptible rats relative to vehicle. In the light-dark box test for unconditioned anxiety, CBD, but not CDPPB, reduced anxiety in Susceptible rats. Resilient rats displayed reduced anxiety in the light-dark box relative to Susceptible rats. Taken together, the present data indicate that enhancement of mGlu5 receptor signaling in populations vulnerable to stress may serve to offset a resistance to fear memory extinction without producing anxiogenic effects. Furthermore, in a susceptible population, CBD attenuates unconditioned but not conditioned fear. Taken together, these findings support the use of predator-threat stress exposure in combination with stress-susceptibility phenotype classification as a model for examining the unique drug response profiles and altered neuronal function that emerge as a consequence of the heterogeneity of psychophysiological response to stress.

## Introduction

Post-traumatic stress disorder (PTSD) develops in a subset of individuals following a traumatic event (Perkonigg et al., 2000). A characteristic feature of PTSD is impaired fear memory extinction (Orr et al., 2000; Guthrie and Bryant, 2006), which contributes to the persistent anxiety and hyperarousal experienced by affected individuals (Herman, 1992; Norrholm et al., 2011). Fear extinction is an active learning process where stimuli that previously elicited fear are repeatedly presented in the absence of threat to produce a gradual reduction in fear response (Bouton et al., 2006). While extinction-based exposure therapies are frequently used as a strategy for treating anxiety-like disorders, PTSD-associated extinction deficits reduce the efficacy of these treatments (Schottenbauer et al., 2008). Consequently, there is a need to improve currently available therapies for PTSD. One approach that directly addresses extinction deficits in PTSD would involve the co-administration of extinction-enhancing pharmacological agents with exposure therapy to improve treatment outcomes (e.g., Rothbaum et al., 2014).

Animal models are essential to interrogate the neurobiology underlying fear extinction and for the development of novel extinction-enhancing therapeutics. The most commonly used models are grounded in Pavlovian fear conditioning principles (Pavlov, 1927; Rescorla, 1988). Fear conditioning involves pairing an unconditioned aversive stimulus (US; e.g., mild electric shock) with neutral conditioned stimuli (CS; e.g., a discrete cue or context) until a conditioned fear response (CR; e.g., freezing, changes in heart rate) is produced following delivery of the CS alone. Like exposure therapy, fear extinction training involves prolonged, or repeated presentations of the CS alone, and ideally results in the gradual elimination of the CR (Rothbaum and Schwartz, 2002; Barad, 2005).

Footshock stress is commonly used to study fear learning, and although these models have contributed substantially to our understanding of neural circuits involved in conditioning and extinction of fear, several alternatives to footshock have been established, each offering unique and complementary contributions to the field. Notably, inescapable exposure to species-relevant predator odors (also termed as predator scent stress, PSS) can evoke persistent alterations in behavioral and physiological response in rats that mirror the symptom profile of fear and anxiety related disorders such as PTSD. Exposure of rodents to 2, 3, 5-Trimethyl-3-thiazoline (TMT), a synthetically derived component of fox feces (Vernet-Maury et al., 1984) induces hyperarousal (Hebb et al., 2003), anxiety (Rosen et al., 2015), social dysfunction (Stockman and McCarthy, 2017), vulnerability to substance use (Schwendt et al., 2018), and contextually cued defensive behaviors (Fendt and Endres, 2008; Homiack et al., 2017), indicating the incidence of both sensitized and conditioned fear and anxiety like behaviors.

A key advantage of using PSS models is the ability to examine physiological features associated with the individual differences in vulnerability to such stress. As previously established for PSS using cat odor (Cohen et al., 2003, 2014; Nalloor et al., 2011), rats can be separated into Susceptible, Resilient and Intermediate phenotypes based on scores in both the elevated plus maze (EPM) and habituation in the acoustic startle response (ASR) 7 days after PSS exposure. Control rats are placed into the PSS context without predator odor and are later assessed in the EPM and ASR. Most humans exposed to trauma initially display symptoms of distress and anxiety which dissipate within 1–4 weeks following the trauma (Foa et al., 2006). A similar pattern is observed in the PSS model: 1 day following PSS exposure, approximately 90% of PSS exposed rats are classified as Susceptible; by the 7th day post-exposure, this rate drops to 25%, nicely paralleling the human condition (Cohen et al., 2003). We and others have demonstrated that in unstressed Control rats, the percent of rats classified as Susceptible is much lower, at 1.33–4% (Cohen et al., 2003; Schwendt et al., 2018). Thus, the anxiety phenotype in Susceptible rats is induced by PSS exposure, and is not present in the absent of such exposure.

Likewise, we have recently reported that a single 10-min exposure to TMT gives rise to distinct stress-Susceptible and Resilient phenotypes in Sprague-Dawley rats, with each group presenting distinct behavioral, hormonal, and molecular signatures (Schwendt et al., 2018). Notably, we found that while all TMT-exposed rats and Control rats displayed similar freezing during the PSS exposure, only Susceptible rats displayed increased freezing upon re-exposure to the PSS context whereas Resilient and Control rats did not. Furthermore, Susceptible rats do not decrease freezing over the course of 5 days of extinction exposures to the PSS context (Schwendt et al., 2018). Taken together, these findings indicate phenotypic heterogeneity among populations of stressed animals which may have an unseen influence on the conclusions gained measuring fear extinction within the entire population of stressed animals. Thus, studies addressing differential vulnerabilities may reveal novel fear-associated adaptations.

In healthy humans, neuroimaging studies have revealed an important role for neural activity in the circuitry encompassing medial prefrontal cortex (mPFC) and amygdala during fear extinction. Increased activity is observed in the ventral medial prefrontal cortex (vmPFC) and decreased activity observed in the dorsal lateral prefrontal cortex (dlPFC; Milad et al., 2007) and amygdala (LaBar et al., 1998). Opposite patterns are demonstrated in humans with PTSD, with low vmPFC activity and high activity in both dlPFC and amygdala (Milad et al., 2009). As noted above, the neural correlates of fear extinction in rodents have been extensively studied using footshock models, and suggest a conserved mechanism also involving functional interactions between the mPFC and amygdala. In the rodent mPFC, the prelimbic (PL) and infralimbic (IL) cortices (analogous to the dlPFC and vmPFC in humans, respectively) are strongly interconnected with the basolateral amygdala (BLA; Hoover and Vertes, 2007). The BLA is required for extinction of conditioned footshock (Falls et al., 1992), and serves to regulate fear response through output to the central amygdala (CeA) and brainstem regions (Royer et al., 1999; Haubensak et al., 2010). Chemogenetic, or electrical stimulation of IL or PL pathways targeting the BLA reveal opposing influences (Herry et al., 2008; Senn et al., 2014), with IL enhancing, and PL impairing extinction (Sierra-Mercado et al., 2011). Additionally, inhibitory and excitatory IL and PL projections (respectively) regulate BLA excitability, stabilizing fear response inhibition (Cho et al., 2013). This evidence suggests that extinction of footshock conditioned fear requires a switch from PL- to IL-mediated reciprocal signaling through the BLA. Indeed, the assessment of neuronal activity using c-Fos immunoreactivity reveals high Fos expression in the IL, but not PL following extinction, and PL and BLA Fos expression correlating with extinction resistance (Knapska and Maren, 2009). While TMT exposure is also associated with changes in amygdala, IL, and PL activity (Sevelinges et al., 2004; Hwa et al., 2019), and recent studies implicate these regions in the extinction of conditioned fear with alternative predator odors, how the coordinated activity across these regions may contribute to the suppression of TMT conditioned fear remains undetermined.

Glutamate receptor signaling has been the focus of many efforts in the development of extinction-enhancing agents. Metabotropic glutamate receptor 5 (mGlu5) subtype regulates bidirectional synaptic plasticity in fear-associated brain regions including the mPFC and BLA (Niswender and Conn, 2010). Pharmacological and genetic inhibition of mGlu5 impairs extinction of both cues and contexts paired with footshock (Xu et al., 2009; Fontanez-Nuin et al., 2011; Sepulveda-Orengo et al., 2013; Sethna and Wang, 2016), and administration of mGlu5 positive allosteric modulators (PAMs) enhances extinction of a footshock-paired context (e.g., Sethna and Wang, 2014). Although antagonism of mGlu5 receptors impairs consolidation of extinction memory, these drugs have also been found to produce anxiolytic effects (Porter et al., 2005; Rahman et al., 2017). The consequences of glutamate receptor modulation on the extinction of predator odor conditioned fear has been assesses in only one study that demonstrated partial agonism of NMDA receptors with D-cycloserine enhanced extinction of a cat odor-paired context (Sarıdoğan et al., 2015). We have previously found increased mGlu5 gene expression in the amygdala and mPFC of Resilient rats following re-exposure to the TMT-associated context (Schwendt et al., 2018). In the same study, daily systemic treatment with the mGlu5 PAM CDPPB during extinction of the TMT-paired context increased freezing in a cohort of Susceptible rats that previously underwent cocaine self-administration (Schwendt et al., 2018). However, as chronic cocaine can alter both function and mGlu5 receptors numbers in brain regions associated with fear signaling (Hao et al., 2010; Ghasemzadeh et al., 2011; Schmidt et al., 2011), a primary goal here was to examine the effects of CDPPB on fear extinction in cocaine-naïve rats using this model.

Like mGlu5, CB1 receptors are abundantly expressed in the BLA and mPFC and are important modulators of fear and anxiety signaling (Chhatwal and Ressler, 2007). Previous studies have revealed dysregulated expression of CB1 receptors and abnormal levels endocannabinoids in subjects with PTSD (Neumeister et al., 2015), as well as in rodent PTSD models (Schwendt et al., 2018). In rodents, genetic or pharmacological inhibition of CB1 receptors impairs extinction (Marsicano et al., 2002), while CB1 agonists have extinction-enhancing effects (Chhatwal et al., 2005; Campolongo et al., 2009). However, CB1 agonists can also produce biphasic anxiogenic and anxiolytic effects (Haller et al., 2004; Sink et al., 2010), which may compromise their clinical usefulness. Several recent studies have demonstrated that cannabidiol (CBD), a component of cannabis which lacks THC-like psychoactive effects (Campos et al., 2012b), may serve to mitigate symptoms of PTSD by increasing extinction and reducing post-trauma anxiety in both humans and rodents (Bitencourt et al., 2008; Das et al., 2013).

Here we evaluated the effects of CDPPB and CBD on the extinction of contextual fear in a PSS model. We focused our investigation on rats with stress-Susceptible phenotype, as Resilient and Control rats do not demonstrate freezing upon re-exposure to the conditioning context (Schwendt et al., 2018). Further, this study explored possible changes in neuronal activity (via Fos expression) produced by fear extinction training within the PL, IL, and BLA regions. Given the involvement of PL, IL, and BLA neuronal activity in extinction to conditioned footshock (Cho et al., 2013), and evidence indicating an important role for mGlu5 receptor function (Sethna and Wang, 2016), we predicted that treatment with CDPPB would (a) enhance extinction of contextual fear, and (b) increase Fos expression in all three regions. Finally, this study also considered the effects of CDPPB and CBD treatment on unconditioned anxiety, as anxiogenic effects may compromise the utility of these drugs for fear-extinction therapies.

## Materials and Methods

### Animals

Adult male Sprague-Dawley rats (Charles River; *N* = 307) were individually housed in ventilated cages in a vivarium maintained on a 12:12 light-dark cycle (lights off at 7:00 am). Prior to the beginning of the study, rats were acclimated to the vivarium for 7 days with *ad libitum* access to food and water. Beginning 72 h after arrival, rats were carefully handled to become familiar with experimenters (always one male and one female experimenter) prior to stress induction. Food access was restricted to 20 g/day from the beginning of testing to be consistent with our previous publication with this model (Schwendt et al., 2018). All procedures were performed within 4 h of the beginning of the dark cycle. Rats arrived in 3 cohorts of 80–110 rats over the course of 1 year. Procedures were approved by the Institutional Animal Care and Use Committee at the University of Florida.

### Drugs

3-Cyano-N-(1,3-diphenyl-1H-pyrazol-5-yl)benzamide (CDPPB, 30 mg/kg; Abcam Biochemical) was suspended in 10% Tween 80 (Sigma-Aldrich) in phosphate-buffered saline (PBS) to a final concentration of 30 mg/ml and injected subcutaneously (s.c.). The dose of CDPPB was based on previous studies indicating an effect on fear extinction (Sethna and Wang, 2014). Cannabidiol (CBD, 5 mg/kg) was provided by the NIDA controlled substances program (RTI, Research Triangle, NC) and dissolved in a mixture of 100% ethanol, Cremophor, and 0.9% NaCl to 5 mg/ml and injected intraperitoneally (i.p.). The dose of CBD was based on previous studies demonstrating an effect on enhancing footshock conditioned contextual fear conditioning (Jurkus et al., 2016), and is within a range of doses found to produce anxiolytic effects (Guimarães et al., 1990). Locomotor testing was not performed as given doses of CDPPB and CBD do not affect locomotion in rats (Gass and Olive, 2009; Ren et al., 2009). 2, 3, 5-Trimethyl-3-thiazoline (TMT, 5 μl; BioSRQ) was presented undiluted (97% purity). The amount of TMT used for predator odor exposures was based on previous studies by our laboratory and others (Tanapat et al., 2001; Day et al., 2004; Schwendt et al., 2018).

### Experimental Procedures

#### Predator-Scent Stress Exposure

**FIGURE 1:**
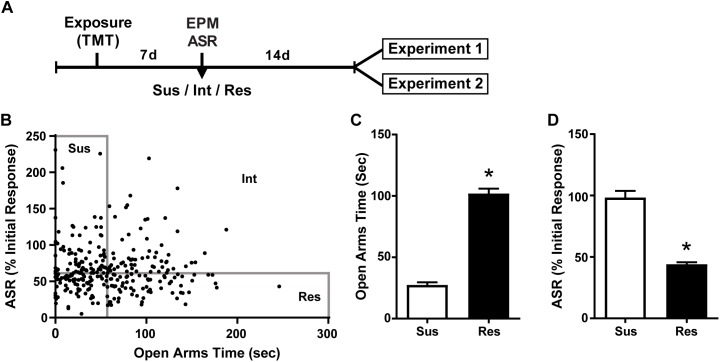
Susceptible and Resilient rats display distinct behavioral phenotypes. **(A)** Timeline for behavioral classification. **(B)** Time spent in the open arms of the EPM plotted against % habituation of acoustic startle response (ASR) for all TMT exposed rats (*n* = 299). Median splits performed on EPM open arm time (median = 56.2 s) and % ASR habituation (median = 61.2%) were used to classify rats into Susceptible (Sus, *n* = 74), Resilient (Res, *n* = 74), or intermediate (Int, *n* = 151) phenotypes. **(C)** Sus rats spent less time in the open arms of the EPM and **(D)** exhibited attenuated habituation to ASR relative to Res rats. ^∗^*p* < 0.0001.

The timeline for the predator-scent stress exposure and assessment of anxiety is shown in Figure 1A. Six to 10 days after arriving in the vivarium, rats received a single exposure to TMT in a covered, clear cylindrical Plexiglas chamber (BioBubble Pets; 40 cm diameter × 35 cm height) with steel mesh flooring above a clear plastic dish. Prior to each session, TMT (5 μl) was placed on a square of filter paper positioned in the center of the dish. Rats were individually placed in the test chamber for a single 10 min exposure. Test chambers were cleaned with 70% ethanol between sessions. Exposures were videotaped.

Power analyses (G power) indicated the number of animals needed was 7–8/treatment group with a significance level of 0.05. We have previously shown that the incidence of the Susceptible phenotype amongst TMT-exposed rats ranges from 14 to 21.8% (Schwendt et al., 2018). Thus, with a target of 8 rats/group for a total of 16 Susceptible rats needed for Experiments 1 and 32 needed for Experiment 2, we initially exposed 307 rats to TMT.

#### Elevated Plus Maze (EPM)

Seven days after TMT exposure, all rats were tested on the EPM according to previously described procedures (Pellow et al., 1985). The EPM apparatus (Med Associates) was made from black acrylic and consists of four arms (50 cm length × 10 cm width) raised 50 cm from the floor. Two open arms (2.5 cm high walls) and two closed arms (50 cm high walls) are joined by a center square platform (10 cm × 10 cm) illuminated at 50 lux. Rats were individually placed on the center platform facing a closed arm and allowed to move freely for 5 min. Sessions were filmed by a camera secured above the maze. The EPM was cleaned with 70% ethanol between tests. Total time spent in the open arms excluding time in the center area (OA time) was recorded with EthoVision XT 14 software (Noldus Information Technology) and served as a measure for anxiety.

#### Acoustic Startle Response

Immediately after EPM testing, habituation of acoustic startle response (ASR) was assessed according to Valsamis and Schmid (2011). Four ventilated soundproof chambers (San Diego Instruments) each contained a transparent plexiglass cylinder that rested on a pressure-sensitive platform. An accelerometer fitted to the platform measured changes in pressure created from movement of the rat’s body, and the maximum response amplitude was registered during presentation of acoustic stimuli. Accelerometer calibration and acoustic sound levels were routinely checked, and chambers were cleaned with CaviCide disinfectant (Metrex) and 70% ethanol between sessions. Rats (four at a time) were secured in the plexiglass cylinders and acclimated to the chamber for 5 min. Next, 30 pulses of 110 db white noise were delivered for 40 ms followed by a variable (30–45 s) intertrial interval. Startle habituation was calculated as the percent change in startle amplitude from the first six trials to the last six trials.

#### Experiment 1 – The Effects of CDPPB of Contextual Fear Extinction and Context-Induced Fos Protein Expression

##### Fear extinction

**FIGURE 2:**
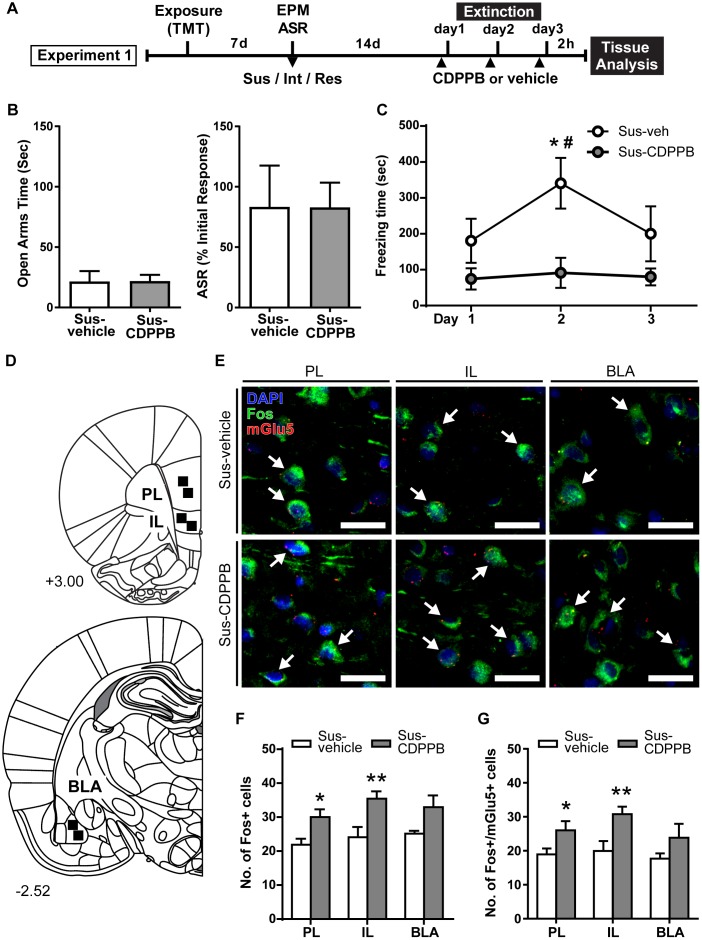
CDPPB reduces context dependent freezing in Sus rats and increases Fos expression in the medial prefrontal cortex. **(A)** Timeline for Experiment 1. **(B)** Treatment groups did not differ in EPM open arm time or % ASR habituation. **(C)** Mean ± SEM of freezing in vehicle (*n* = 7) or CDPPB (*n* = 7) treated Susc rats during 10 min extinction sessions in the TMT context initiated 14 days after phenotype classification; CDPPB reduced freezing on day 2 of extinction. **(D)** Schematic indicating the regions of interest (ROI) used for characterization of extinction dependent neuronal activity in the prefrontal cortex (top) and basal lateral amygdala (BLA; bottom). **(E)** Representative confocal images from the selected ROIs in the prelimbic cortex (PL; left), infralimbic cortex (IL; middle) and BLA (right) showing fluorescent immunolabeling of Fos (green) protein, labeling of mRNA for mGlu5 using fluorescent *in situ* hybridization, and DAPI (blue) nuclear staining, in vehicle (top panel) and CDPPB (bottom panel) treated susceptible rats; scale bar: 20 μm, 63× magnification. Fos+mGlu5 co-expression is indicated with white arrows. **(F)** CDPPB increased the number of Fos labeled cells in the PL and IL regions on day 3 of extinction. **(G)** The effects of CDPPB on the number of Fos+/mGlu5+ double-labeled cells in the PL, IL, and BLA on day 3 of extinction. ^∗^*p* < 0.05, ^∗∗^*p* < 0.01 relative to vehicle, ^#^*p* < 0.05 relative to Days 1 and 3.

The timeline for this experiment is shown in Figure 2A. Rats first underwent predator stress induction and EPM/ASR assessment (see above and Figure 1A). Rats classified as Susceptible were randomly subdivided into two groups: Sus-Veh and Sus-CDPP (*n* = 7/group). Two weeks after anxiety assessment, rats underwent three contextual fear extinction sessions on three separate days. We previously found that in Susceptible rats with a history of cocaine self-administration, treatment with CDPPB immediately prior to placement into the TMT context increased freezing on Days 2–4 of extinction (Schwendt et al., 2018). Here we sacrificed rats immediately after Day 3 of extinction in order to examine neuronal activity during the last extinction session. Twenty minutes before sessions, rats were injected with either vehicle or CDPPB and returned to their home cage. Rats were then placed into the exposure chamber in the absence of TMT for 10-min sessions (one session/day). To avoid potential residual scent, we used plexiglass chambers which never had contact with TMT. Sessions were filmed, and freezing was quantified offline using the mobility detection function in Ethovision XT 14 software according to Pham et al. (2009). Freezing in rats is a species-specific threat-related defensive strategy that is defined by the absence of movement except for respiration (Fanselow, 1980).

##### Combined Fos immunohistochemistry and fluorescent *in situ* hybridization

Next, we performed fluorescent immunolabeling for Fos protein to measure neuronal activity during the third extinction session. Fos is widely used relative marker for neuronal activation (Lanahan and Worley, 1998; Kovács, 2008). As previous studies have demonstrated a role for mGlu5 in the regulation of cFos, and stimulation of mGlu5 with CDPPB can enhance cFos expression (Mao and Wang, 2003; Mao et al., 2005; Uslaner et al., 2009), we also performed dual-labeling for mGlu5 mRNA. Two hours after the third extinction session, rats were administered an overdose of sodium pentobarbital (Euthasol, 1 ml, i.p.) and transcardially perfused in nuclease free 0.9% NaCl followed by cold 4% paraformaldehyde (PFA) in PBS. Brains were removed, post-fixed for 12 h at 4°C in 4% PFA in PBS, cryopreserved in 30% sucrose in PBS, frozen and kept at -80°C until sectioning. Twelve-μm-thick tissue sections corresponding to the rat PL/IL (+3.00 mm relative to Bregma) and BLA (-2.52 mm) according to rat brain atlas (Paxinos and Watson, 2005), were collected using a freezing cryostat (Leica CM1950). Tissue sections were direct-mounted onto Superfrost Plus Gold slides (Fisher Scientific), dried, and stored at -80°C. Fluorescent *in situ* hybridization of *GRM5* (mGlu5) mRNA was performed using the RNAscope Multiplex Fluorescent Reagent Kit (ACDBio) according to the manufacturer’s instructions with a few modifications (Wang and Zhuo, 2012). Slides were first equilibrated to room temperature (RT) before heating to 60°C for 45 min. Next, sections were fixed in cold 4% PFA in PBS and dehydrated using ethanol gradient of 50, 70, 100, and 100% in consecutive 5 min incubations. Slides were boiled in target retrieval reagent (ACDbio), washed in nuclease-free H_2_O, and again dehydrated in 100% ethanol. Proteinase digestion of sections was conducted using pretreatment #3 (ACDbio) at 40°C for 30 min under humidity-controlled conditions (HybEZ hybridization oven; ACDbio). The RNAscope probe for *GRM5* (ACDBio: 471241-C2, lot 17335A) was diluted with C1 diluent probe (1:50) and applied to sections. Slides were then incubated 2 h at 40°C for hybridization of probe to target mRNAs. Signal amplification was performed with preamplifier and amplifier probes at 40°C (AMP 1, 30 min; AMP 2, 15 min; AMP 3, 30 min; AMP 4, 15 min). For AMP4 (15 min), Alt-A was selected so that the target probe could be detected with ATTO 550 (Cy3) fluorescent label. Immediately following *in situ* hybridization, slides were rinsed three times in Tris-buffered saline (TBS) and blocked (0.3% Triton x-100 and 5% NGS in TBS) for 1 h at RT. TBS was used for all rinses and antibody dilutions. Sections were incubated in rabbit anti-Fos antibody (1:1000; Synaptic Systems) overnight at 4°C. The next day slides were rinsed and then incubated in goat anti-rabbit Alexa 594 secondary antibody (1:1000; Invitrogen) for 2 h at RT. Finally, sections were rinsed again before counterstaining with DAPI (Invitrogen) and coverslipped using ProLong Gold antifade mounting reagent (Invitrogen).

##### Imaging

Fluorescent images were captured using Zeiss LSM70 inverted Axio-Observer 3-channel spectral confocal microscope and Zen 2012 software. Multitrack sequential imaging settings were applied to avoid inter-channel crosstalk effects. The 405, 488, and 561 nm laser lines were used for excitation of DAPI, Fos (Cy3), and mGlu5 (FITC). Two regions of interest were selected for each brain area (PL, IL, and BLA; Figure 2D) and *Z*-stacks were acquired at 1 μm intervals using a 63× oil immersion objective. Image stacks were imported into NIH Image J software (Schneider et al., 2012) and analyzed offline. Only the middle five focal planes from each *Z*-stack were used for analysis. To measure co-expression, *Z*-stacks were first converted to composite images for separation of individual color channels. The red channel (mGlu5) was then turned off, and cells containing green (Fos) staining were marked using the NIH Image J multi-point tool on each focal plane. Then, the green channel was then turned off, and the red channel was used to mark mGlu5 puncta in the same manner. Individual cells were distinguished based on DAPI nuclei staining. Fos expression was established if staining within a cell was detectable on three consecutive focal planes, and mGlu5 expression was established if a cell contained mRNA puncta on three consecutive focal planes. Under these conditions, cells expressing both Fos and mGlu5 were considered positive for co-expression. The total number of Fos expressing and Fos/mGlu5 co-expressing cells were counted for each selected area. The average number of cells for each parameter across the two selected areas was calculated for the three brain regions for each rat.

#### Experiment 2 – The Effects of CDPPB (or CBD) on Unconditioned Anxiety and Contextual Fear Extinction

**FIGURE 3:**
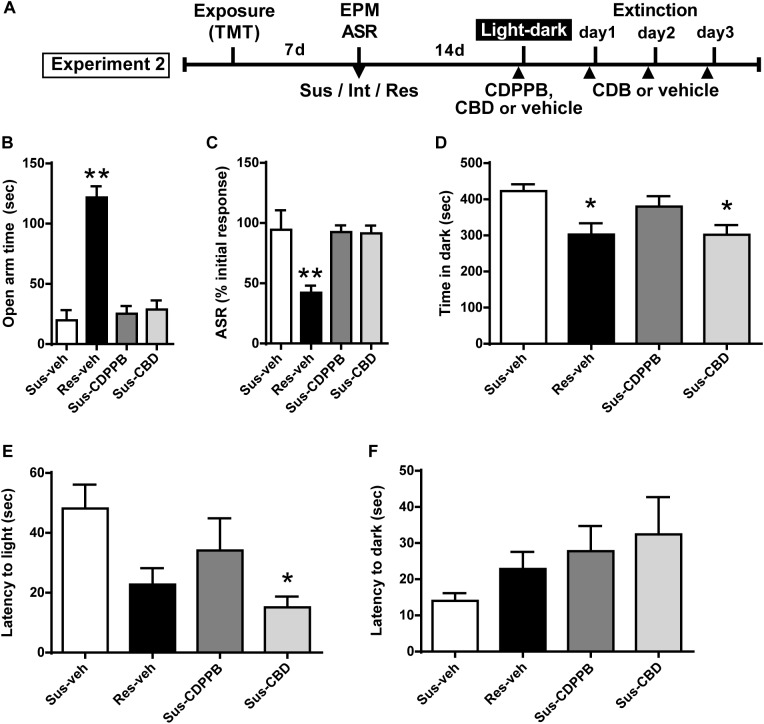
Cannabidiol attenuates increased anxiety-like behavior in the light-dark box test in Susceptible rats. **(A)** Timeline for Experiment 2. **(B)** Vehicle (Sus-veh, *n* = 8), CDPPB (Sus-CDPPB), and CBD (Sus-CBD, *n* = 8) treated Sus rats spent less time in the open arms of the EPM and **(C)** exhibited reduced habituation to ASR relative to vehicle treated Res rats (Res-veh, *n* = 8). **(D)** CBD treated Sus rats and Res rats spent less time in the dark-box relative to vehicle treated Sus rats. **(E)** CBD treatment reduced the latency to enter the light-box in Sus rats. **(F)** No differences were observed in latency to enter the dark-box. ^∗∗^*p* < 0.05 relative to Res-veh; ^∗^*p* < 0.05 relative to Sus-veh.

The timeline for this experiment is shown in Figure 3A, 4A. Rats first underwent predator stress induction and anxiety assessment as described above. Susceptible rats were randomly selected from the sample to create vehicle (Sus-Veh; *n* = 8), CDPPB treated (Sus-CDPPB; *n* = 8), and CBD treated (Sus-CBD; *n* = 8) groups. Resilient rats were treated with vehicle(Res-Veh; *n* = 8).

**FIGURE 4:**
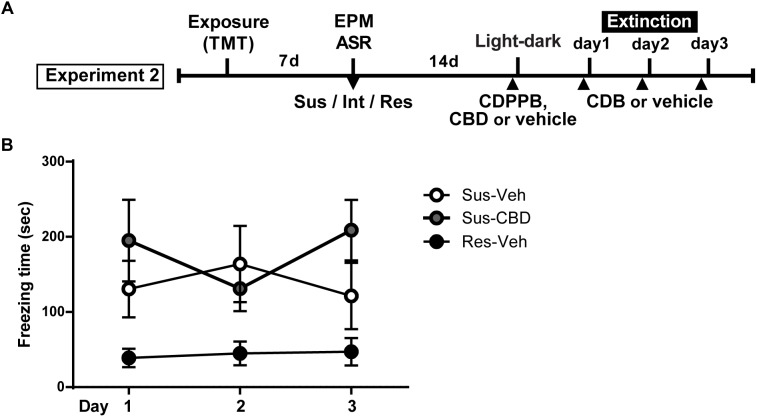
Cannabidiol does not reduce increased context dependent freezing in Susceptible rats. **(A)** Timeline for Experiment 2. **(B)** Mean ± SEM of freezing in vehicle (Sus-veh; *n* = 8) and CBD (Sus-CBD; *n* = 8) treated Sus rats and vehicle treated Res rats (Res-veh; *n* = 7) during 10 min extinction sessions. A main effect of Group was detected with Sus-veh and Sus-CBD displaying increased freezing relative to Res-veh rats.

##### Light-dark box

Two weeks after anxiety assessment and phenotype classification, rats underwent light-dark box testing based on previously described procedures (Crawley, 1981). Light-dark box apparatus consisted of a plexiglass box with two separate compartments of equal dimensions (40 cm × 44 cm × 37 cm); a light compartment (light box) with translucent walls illuminated at 300 lux, and a dark compartment (dark box) with blackened opaque walls. An opening in the dividing wall allowed free movement of rats between the compartments. Prior to testing, rats were treated with vehicle, CDPPB, or CBD in their home cage. Following pretreatment (CDPPB: 20 min; CBD: 30 min), each animal was individually placed in the center of the light box and allowed to roam freely for 10 min. Sessions were filmed, and the following behaviors were hand scored by an experimenter blind to the conditions: (1) latency to enter the dark box from the light box and vice versa; (2) the number of transitions between compartments; and (3) the duration of individual compartment visits. The apparatus was cleaned with 70% ethanol between trials.

##### Fear extinction

Next, rats were used to assess differences between Resilient and Susceptible rats in fear extinction and the ability of CBD to enhance the extinction of TMT conditioned fear. The day after light-dark box testing, rats were administered vehicle or CBD 20 min prior to testing (Figure 4A). Rats received the identical treatment that they had received on the day prior for light-dark box testing. Procedures for fear extinction were identical to Experiment 1.

### Data Analysis

GraphPad Prism (version 6.0) was used for statistical analysis with the alpha level set at *p* ≤ 0.05. Unpaired *t*-tests were used to compare phenotypic differences in EPM and ASR behavior for rats used in Experiments 1–2. Unpaired *t*-tests were also used to test for within region differences in Fos expression between treatment groups. One-way analyses of variance (ANOVAs) were used to assess differences in EPM and ASR behavior for rats later treated with vehicle, CBD or CDPPB to ensure no pre-existing differences prior to initiation of testing and pharmacological treatment. Freezing during fear extinction was analyzed by two-way mixed factorial repeated-measures (RM) ANOVAs with Treatment as the between-subjects factor and Day as a within-subjects factor. Significant interactions were followed by Tukey’s *post hoc* analyses with corrections for multiple comparisons.

## Results

### Predator Odor Stress and Susceptibility Classification

A total of 307 rats were exposed to TMT. ASR data files for 8 rats were corrupted and thus the following calculations are based on the data from 299 rats. The median time spent in the OA of the EPM was 56.2 s and median habituation of the ASR was 61.2%. Rats that fell below the median for time spent in the OA and above the median for ASR habituation were classified as Susceptible as in our previous report (Schwendt et al., 2018). Rats were classified as Resilient if they fell above the median for time spent in the OA and below the median for ASR habituation. This resulted in 74 rats (25%) meeting criteria for the Susceptible phenotype and an equal number meeting criteria for the Resilient phenotype (Figure 1B). A total of 46 Susceptible rats and 8 Resilient rats were used for the present set of experiments. More Susceptible and Resilient rats were generated than were needed for the present set of experiments and were used for other experiments to be published at a later date. Rats not classified as either Susceptible or Resilient (Intermediate; *n* = 151) were eliminated from the experiment. Susceptible (Sus) rats spent less time in the open arms of the EPM [*t*(26) = 4.482, *p* < 0.0001, Figure 1C], and exhibited less reductions in ASR magnitude [*t*(26) = 4.515, *p* < 0.0001, Figure 1D] compared to Resilient rats. Freezing was assessed on the day of TMT exposure in a subset of Susceptible (*n* = 20) and Resilient (*n* = 20) rats; no phenotypic differences in freezing were found (mean ± SEM: Susceptible 10.2 ± 2.438 s; Resilient 10.8 ± 2.624 s).

### Experiment 1

Rats were assigned to receive vehicle (Sus-vehicle, *n* = 7) or CDPPB (30 mg/kg, s.c.; Sus-CDPPB, *n* = 7) prior to extinction sessions such that there were no differences in prior EPM and ASR scores between treatment groups (Figure 2B). To examine the effects of CDPPB on fear extinction, a 2-way RM ANOVA conducted on freezing behavior (Figure 2C) revealed a main effect of Treatment [*F*(1,11) = 6.803, *p* = 0.024] and Day [*F*(2,22) = 3.905, *p* = 0.035], and a significant Treatment × Day interaction [*F*(2,22) = 5.134, *p* = 0.015]. *Post hoc* tests revealed that on Day 2, Sus-CDPPB rats froze significantly less than Sus-veh rats, and no between-group differences in behavior were observed on Days 1 or 3. Within the Sus-veh group, Day 2 freezing was greater than Days 1 and 3 (Figure 2C). Analysis of Fos immunoreactive cells revealed increased Fos expression in the PL [*t*(10) = 2.80, *p* = 0.02], IL [*t*(10) = 3.03, *p* = 0.01] of Sus-CDPPB rats compared to Sus-veh (Figure 2F). In the BLA, a trend toward increased Fos protein expression was detected in Sus-CDPPB group (*p* = 0.054). Co-expression of Fos and mGlu5 was abundant across analyzed regions of both treatment groups, however, the level of Fos/mGlu5 expression overlap varied between regions (Sus-veh, [*F*(2,15) = 2.756, *p* = 0.0125]; Sus-CDPPB [*F*(2,15) = 0.830, *p* < 0.0001]), with lower co-expression in the BLA (∼70%) compared to both IL and PL regions (∼90%; data not shown). Perhaps due to this high degree of co-localization, CDPPB treatment increased Fos expression in mGlu5-positive cells in a manner that was similar to overall Fos expression: expression was increased in the PL [*t*(10) = 2.92, *p* = 0.04] and IL [*t*(10) = 3.30, *p* = 0.01], but not in the BLA (Figure 2G). Representative images of dual Fos/mGlu5 labeling from the regions depicted in Figure 2D are shown in Figure 2E.

### Experiment 2

Subsets of Susceptible (Sus) and Resilient (Res) rats were used to generate four treatment groups: Sus-veh, Sus-CDPPB, Sus-CBD, and Res-vehicle. Examining only the rats utilized for Experiment 2, a one-way ANOVA revealed phenotypic differences in EPM open arm time [*F*(3,27) = 37.61, *p* < 0.0001, Figure 3B] and ASR habituation [*F*(3,27) = 8.028, *p* = 0.0006]. All three groups of Sus rats spent less time compared to Res rats in the open arms of the EPM (Sus-veh, *p* < 0.0001; Sus-CDPPB, *p* < 0.0001; Sus-CBD, *p* < 0.0001, Figure 3B), and exhibited less habituation of the ASR relative to Res rats (Sus-veh, *p* = 0.0023; Sus-CDPPB, *p* = 0.0023; Sus-CBD, *p* = 0.0029, Figure 3C). In the light-dark box test, Group differences in time spent in the dark side were found [*F*(3,27) = 4.686, *p* = 0.0092, Figure 3D]. *Post hoc* analysis revealed Sus-veh rats spent more time in the dark side compared to Res-veh, indicating the preservation of anxiety phenotypes 2 weeks after classification with EPM and ASR. In Susceptible rats, CDPPB did not alter time spent in the dark (*p* = 0.692, Sus-CDPPB vs. Sus-veh), however, CBD had an anxiolytic effect, demonstrated by a reduction in dark box time (*p* = 0.023, Sus-CDB vs. Sus-veh). Time spent in the dark in Sus-CBD rats did not differ from Res-veh rats. One-way ANOVA also revealed differences in latency to enter the light box [*F*(3,25) = 3.315, *p* = 0.036, Figure 3E], with CDPPB again having no effect, and CBD reducing the time to enter relative to the Sus-veh treated group. No phenotypic or treatment differences were observed in the latency to enter the dark side (Figure 3F).

On the day following the light-dark box test, rats began fear extinction trials that continued for 3 days. Prior to each trial animals received CBD (5 mg/kg, i.p.) or vehicle. A two-way repeated measures ANOVA on time spent freezing revealed a significant main effect of group [*F*(2,20) = 4.106, *p* = 0.032] and a trend toward a significant group × time interaction [*F*(4,40) = 2.197, *p* = 0.08]. There was no main effect of time [*F*(2,40) = 0.2389, n.s.] (Figure 4B).

## Discussion

Here we found that 7 days after a single exposure to the predator odor TMT, populations of stress Susceptible and Resilient rats emerged following phenotype classification using EPM and ASR scores. Similar to previous studies (e.g., Cohen and Zohar, 2004), including our own (Schwendt et al., 2018), 25% of rats met criteria for the Susceptible phenotype and an equal number met criteria for the Resilient phenotype. Moreover, we found that a majority of rats displayed EPM and ASR behavior intermediate between Susceptible and Resilient rats, suggesting that the two phenotypes were indeed representative of extremes of the susceptibility spectrum. In the current study, light-dark box testing 2 weeks after the initial classification revealed that un-conditioned anxiety also differs between Susceptible and Resilient phenotypes.

Consistent with our previous report (Schwendt et al., 2018), the results of Experiment 2 indicate that 3 weeks following the initial TMT context pairing, conditioned fear in Susceptible rats was greater than in Resilient rats. While some previous studies failed to produce a conditioned fear response with TMT (McGregor et al., 2002; Blanchard et al., 2003; Rosen, 2004), these studies only tested for such a response 24 h after TMT exposure. We hypothesize that conditioned freezing in the TMT-paired context here and in our previous report (Schwendt et al., 2018) may be due to the inclusion of a post-stress incubation period. A lengthier exposure time in a medium size chamber may also have contributed to an accumulation of contextual CS information sufficient to generate a defensive response upon re-entry to the TMT-paired context; exposures in smaller chambers do not have this effect (Takahashi et al., 2008). Furthermore, past studies examining TMT fear conditioning have not distinguished stress-Susceptible rodents from Resilient (Wallace and Rosen, 2000; McGregor et al., 2002; Blanchard et al., 2003), hence any conditioning effects may have been obscured.

Consistent with our previous report, we observed an increase in freezing in vehicle-treated Susceptible rats from Day 1 of extinction to Day 2 (Schwendt et al., 2018). CDPPB treated rats demonstrated less freezing on the 2nd day of re-exposure compared to vehicle rats. One potential interpretation of these data is that vehicle-treated rats experienced a potentiation of fear memory reconsolidation on Day 1 that resulted in greater freezing on Day 2; CDPPB prevented this fear memory reconsolidation, yielding reduced freezing. Alternatively, CDPPB treatment may have aided in extinction memory consolidation on Day 1 by enhancing mGlu5 receptor activity within extinction associated neuronal pathways, manifesting as a decrease in freezing on Day 2. As freezing decreased in vehicle-treated rats from Days 2 to 3, these rats may have engaged in extinction acquisition and consolidation during day 2 which was exhibited on Day 3. Extinction of conditioned fear cannot be said to have occurred in the CDPPB-treated group, as no differences in freezing were observed in this group across days, potentially due to a floor effect on freezing on Day 1. Assessment of freezing in CDPPB-treated rats on a subsequent “recall” test conducted in the absence of CDPPB would potentially have shed some light on whether CDPPB is acting acutely to enhance extinction or fear; however, we had designed the present experiment to assess Fos expression on Day 3. Thus, future work will aim to understand whether CDPPB is reducing freezing through the acquisition, consolidation, or expression of fear extinction behavior by administering CDPPB in different regimens, such as immediately after the re-exposure session or only on Day 1 of extinction.

A reduction in conditioned fear in CDPPB-treated rats is in contrast to our previous report that this dose of CDPPB increases freezing when administered prior to fear extinction in rats with a history of cocaine self-administration. It can be argued that chronic self-administration of cocaine altered the neurobiology underlying the extinction of conditioned fear. Alternatively, previous studies in rats and humans have demonstrated bi-phasic effects of the NMDA receptor partial agonist D-cycloserine, which can either produce a weakening or a strengthening of fear memory contingent upon an individual’s experience during the extinction session (Orr et al., 2000; Bolkan and Lattal, 2014). Furthermore, in cocaine users, D-cycloserine *prevents* the reductions in craving and brain activation produced by extinction of cocaine-associated cues. Taken together, this suggests that cocaine alters the role of glutamate receptors in mediating extinction of both fear and cocaine-associated cues (Price et al., 2013; Prisciandaro et al., 2013). This should be considered in the treatment of anxiety disorders in patients with cocaine use disorder.

A substantial literature devoted to understanding signaling dynamics involved in the extinction of footshock conditioned fear indicates implicates roles of the IL and PL. Indeed, many studies examining expression of neuronal activity markers in these regions have correlated activity in the PL with freezing, and IL activity with reductions in freezing during extinction (Herry et al., 2008; Cho et al., 2013; Senn et al., 2014). Here we found increased Fos activation in CDPPB-treated rats in the absence of significant decreases in freezing on the day that the tissue was collected. It is possible that although extinction may have been occurring in both treatment groups on Day 3, the timing of tissue collection did not permit the assessments of the temporal patterns of PL and IL activity during the session. For instance, we cannot determine whether a brief fear recall event at the beginning of Day 3 extinction produced a depolarization of PL cells which may no longer have been firing near the end of the session. Analysis of Fos+mGlu5 co-expression revealed that the increased Fos expression was most likely a result of CDPPB binding, due to the fact that (a) very few Fos-positive cells did not contain mGlu5 mRNA, and (b) increased Fos expression in mGlu5-positive cells mirrored overall Fos protein increase. Thus, it is possible that the increase in Fos expression is an artifact of CDPPB that is not related to freezing behavior or extinction. It may also possible be the case that CDPPB treatment affected non-extinction associated mGlu5 containing cells. In order to understand the role of this circuitry in contextual fear extinction in Susceptible rats, future work will infuse mGlu5-targeting drugs directly into the mPFC and BLA prior to or after fear extinction sessions.

While mGlu5 receptors represent a promising target for reducing conditioned fear, mGlu5 agonists can produce anxiogenic effects under certain conditions (De Jesús-Burgos et al., 2016; Rahman et al., 2017). Therefore, we evaluated the ability of CDPBB to alter unconditioned fear in the light-dark box. We found that CDPPB neither attenuated nor enhanced anxiety in the light-dark box task. This is in contrast to a recent report that the same dose of CDPPB increased anxiety measures in the light-dark box in unstressed mice and in mice that had consumed alcohol (Lee et al., 2018). Thus, CDDPB or other mGlu5 PAMs may be beneficial in a PTSD population as they would reduce conditioned fear without inducing general anxiety, but caution should be exerted in alcohol users.

Although prior studies in heterogeneous rodent populations have observed extinction enhancing properties of CBD in a conditioned footshock model (Bitencourt et al., 2008; Stern et al., 2012; Do Monte et al., 2013), here there was no effect of CBD on conditioned fear in Susceptible rats. It is possible that TMT exposure was not sufficient to evoke the neuroadaptations necessary for CBD-mediated effects. Indeed, one recent study demonstrated that while CBD enhanced extinction in footshock conditioned rats, extinction was unaffected by CBD in rats administered a reduced number of shocks (Song et al., 2016). Although the dose of CBD used in the current study (5 mg/kg) has been previously demonstrated as effective in mitigating the response to contextually conditioned fear (e.g., Jurkus et al., 2016), this dose is at the lower end of an inverted U-shaped response curve for extinction-enhancing effects, which ranges from 3 to 30 mg/kg (Stern et al., 2012). Here, we utilized a lower dose because higher doses of CBD can also produce anxiogenic effects (Song et al., 2016; Lee et al., 2017). The mechanisms by which CBD exerts pro-extinction and anti-anxiety effects are multifaceted, however, several studies have demonstrated a capacity for indirect potentiation of CB1 receptors (Bisogno et al., 2001; Campos et al., 2013). Endocannabinoid (eCB) signaling is implicated in fear extinction through its involvement in synaptic plasticity (Heifets and Castillo, 2009), and dysregulated eCB signaling has been implicated in PTSD (Neumeister et al., 2013). In addition, CBD can function as a partial agonist of the 5-HT1a receptor (Russo et al., 2005; Campos et al., 2012a; Fogaça et al., 2014). Abnormal 5-HT signaling contributes to PTSD like symptoms (Zhao et al., 2017), and is a target for treatment of the disorder (Sullivan and Neria, 2009). Thus, further study is needed to more exhaustively characterize the potential of CBD in PTSD treatment. While CBD did not enhance extinction, it was effective in reducing anxiety in the light-dark box test. Our findings are consistent with previous work showing benefits of CBD in alleviating heightened anxiety in animals that have experienced prior footshock or restraint-stress (O’Brien et al., 2013; Fogaça et al., 2014; Song et al., 2016). While chronic CBD administration following exposure to a live predator was effective in mitigating predator-induced anxiety 1 week later (Campos et al., 2012a), we are the first to show an immediate effect of CBD in a population of Susceptible rats after only a single dose.

## Conclusion

Here we found evidence that mGlu5 PAMs, such as CDPPB, represent a potential treatment strategy for the reduction of conditioned fear in a PTSD population, an effect possibly mediated by an activation of the PL/IL circuitry. However, some preclinical work (Lee et al., 2018; Schwendt et al., 2018) suggests that caution should be exerted when using this class of drugs in patients with a history of cocaine or alcohol use, as it may inhibit, rather than facilitate extinction. Finally, while CBD produced anxiolytic effects in response to unconditioned fear, it failed to enhance the extinction of conditioned fear in a Susceptible, PTSD-like population of rats. As our results indicate distinct roles for these drugs in unconditioned vs. conditioned anxiety, future consideration should be given to polytherapy with both CBD and mGlu5 PAMs for the enhancement of extinction and relief of anxiety that accompany PTSD. For example, the combination of CBD and an mGlu5 PAM such as CDPPB should be administered for the assessment of unconditioned and conditioned fear.

## Ethics Statement

Procedures were approved by the Institutional Animal Care and Use Committee at the University of Florida.

## Author Contributions

JS, MS, and LK involved in the conception and design of the study. JS, PH, MR, and AB involved in the acquisition of data. JS, AB, LK, and MS involved in analysis and interpretation of the data. JS drafted the manuscript. LK and MS made critical revisions. JS, PH, AB, MR, LK, and MS approved the final version of the manuscript to be published.

## Conflict of Interest Statement

The authors declare that the research was conducted in the absence of any commercial or financial relationships that could be construed as a potential conflict of interest.
